# Olfactory rule learning-induced enhancement in intrinsic neuronal excitability is maintained by shutdown of the cholinergic M-current

**DOI:** 10.3389/fncel.2022.934838

**Published:** 2022-09-29

**Authors:** Richa Awasthi, Naveen Chandra, Edi Barkai

**Affiliations:** Sagol Department of Neurobiology, Faculty of Natural Sciences, University of Haifa, Haifa, Israel

**Keywords:** olfactory rule learning, piriform cortex, pyramidal neurons, intrinsic neuronal excitability, slow afterhyperpolarization, cholinergic modulation

## Abstract

Training rats in a particularly difficult olfactory discrimination task initiates a period of accelerated learning, manifested as a dramatic increase in the rats' capacity to discriminate between pairs of odors once they have learned the discrimination task, implying that rule learning has taken place. At the cellular biophysical level, rule learning is maintained by reduction in the conductance of the slow current (sI_AHP_) simultaneously in most piriform cortex layer II pyramidal neurons. Such sI_AHP_ reduction is expressed in attenuation of the post-burst afterhyperpolarization (AHP) potential and thus in enhanced repetitive action potential firing. Previous studies have shown that a causal relationship exists between long-lasting post-burst AHP reduction and rule learning. A specific channel through which the sI_AHP_ flows has not been identified. The sI_AHP_ in pyramidal cells is critically dependent on membrane phosphatidylinositol 4,5-bisphosphate [PtdIns(4,5)P(2)]. PtdIns(4,5)P(2) regulates the calcium sensitivity of the sI_AHP_ by acting downstream from the rise in intracellular calcium. These findings led to the interesting hypothesis that PtdIns(4,5)P(2) activates a variety of potassium channels. Thus, the sI_AHP_ would not represent a unitary ionic current but the embodiment of a generalized potassium channel gating mechanism. We thus hypothesized that the learning-induced increase in intrinsic excitability is mediated by reduced conductance of one or more of the currents that contribute to the sI_AHP_. Here we first show, using current-clamp recordings, that the post-burst AHP in piriform cortex pyramidal neurons is also mediated by the I_h_, and the contribution of this current to the post-burst AHP is also affected by learning. We also show, using whole-cell patch-clamp recordings, that the sI_AHP_ in neurons from trained rats is not sensitive to blocking membrane phosphatidylinositol 4,5-bisphosphate [PtdIns(4,5)P(2)], and to the blocking of the current mediated by the cholinergic muscarinic acetylcholine receptor (M-current). Further current-clamp recordings also show that blocking PtdIns(4,5)P(2) synthesis and application of a specific IKCa blocker have no effect on the post-burst AHP in neurons from trained as well as control rats. Taken together with results from our previous studies, these data suggest that rule learning-induced long-lasting enhancement in intrinsic neuronal excitability results from reduced conductance of the M-current and thus the slow potassium currents, which control repetitive spike firing.

## Intoduction

### Rule learning

The ability to extract generalizable rules from specific experiences is a fundamental attribute of higher cognitive functioning. Rule learning is a term that describes the phenomenon of *learning how to learn* (also termed meta-learning). Training rats in a particularly difficult olfactory discrimination (OD) task initiates a period of accelerated learning of other odors, manifested as a dramatic increase in the rats' capacity to acquire memories for new odors once they have learned the first discrimination task, implying that rule learning has taken place (Saar and Barkai, 2003, 2009; Chandra and Barkai, 2018).

### Rule learning-induced enhancement in neuronal excitability

Learning-related cellular changes in neurons include modifications at synapses and modifications in the intrinsic properties of neurons. While it is commonly agreed that changes in the strength of connections between neurons in the relevant networks underlie memory storage, an increasing amount of evidence shows that modifications in intrinsic neuronal excitability play a key role in learning-related behavioral changes. Learning-induced enhancement in neuronal excitability has been shown in piriform cortex pyramidal neurons following operant conditioning (Saar et al., 1998, 2001; Saar and Barkai, 2003). This effect is also apparent in hippocampal neurons following classical conditioning of the trace eyeblink response (Moyer et al., 1996; Thompson et al., 1996), the Morris water maze task (Oh et al., 2003), and OD learning (Zelcer et al., 2006). Such enhanced excitability is manifested in reduced spike frequency adaptation in response to prolonged depolarizing current applications (Moyer et al., 1996; Thompson et al., 1996; Saar et al., 2001). Neuronal adaptation is modulated by medium and slow afterhyperpolarization (AHPs), generated by potassium currents, which develop following a burst of action potentials (Madison and Nicoll, 1984; Constanti and Sim, 1987; Schwindt et al., 1988; Saar et al., 2001). Indeed, it was shown in hippocampal and piriform cortex pyramidal neurons that the post-burst AHP amplitude is reduced after learning (Moyer et al., 1996; Saar et al., 1998). OD rule learning results in reduced AHP throughout the population of layer II pyramidal neurons in the piriform cortex. Accordingly, neurons from trained rats generate more action potentials, in response to prolonged intrinsically applied stimulation (Saar et al., 2001).

### Long-lasting AHP reduction is maintained by blocking slow potassium channel(s)

Learning-induced long-term AHP reduction and enhanced excitability are mediated by reduction in the conductance of the slow calcium-dependent potassium current (sI_AHP_), in both the piriform cortex (Saar et al., 2001; Brosh et al., 2006) and hippocampus (Sanchez-Andres and Alkon, 1991; Power et al., 2002). The current reduced by learning is acetylcholine (ACh)-sensitive (Disterhoft et al., 1999; Saar et al., 2001). Olfactory discrimination learning-induced AHP reduction in the piriform is also PKC- and ERK-dependent (Cohen-Matsliah et al., 2007). These sensitivities for ACh and second messenger systems are essential for identifying the channel(s) that is(are) affected by learning since the sI_AHP_ has been suggested to be mediated by more than one channel. Notably, long-term reduction in post-burst AHP, induced *in vitro* by repetitive synaptic activation, requires protein synthesis (Cohen-Matsliah et al., 2010). Results of our previous study support the hypothesis that rule learning induces the reduction of acetylcholine-sensitive potassium current (Saar et al., 2001). Notably, in these previous studies, neurons were recorded in the current-clamp mode, thus not allowing direct measurements of specific currents.

### Rule learning-induced AHP reduction is mediated by metabotropic activation of the GluK2 kainate receptor

It has been previously shown that a brief kainate application or a brief repetitive synaptic stimulation results in a long-lasting reduction of the sl_AHP_ (Fisahn et al., 2005; Melyan and Wheal, 2011). This effect is mediated by activation of the GluK2 (also termed GluR6, when using the older nomenclature) subtype glutamate receptor (Melyan et al., 2002, 2004; Fisahn et al., 2005; Melyan and Wheal, 2011). This metabotropic GluK2 function is distinct from its direct ionotropic action on synaptic transmission (Ruiz et al., 2005).

Using the complex OD task, we showed that brief activation of the GluK2 subtype glutamate receptor, by direct kainate application or repetitive synaptic stimulation, results in long-lasting enhancement of neuronal excitability in piriform cortex neurons from controls, but not from trained rats (Chandra et al., 2019). Importantly, intrinsic neuronal excitability cannot be modulated by synaptic activation in neurons from GluK2 knockout mice (Chandra et al., 2019).

### GluK2 is both necessary and sufficient for OD rule learning

At the behavioral level, we showed that the GluK2 receptor is necessary and sufficient for OD rule learning. We first showed that GluK2 knockout mice are incapable of learning the complex OD task, although their ability to learn simple olfactory tasks (such that do require learning of a rule) remains intact (Chandra et al., 2019). Moreover, viral-induced overexpression of Gluk2 in piriform cortex pyramidal neurons enhances rule learning remarkably. It is also worthwhile noting that humans lacking the gene for GluK2 suffer from mental retardation (Motazacker et al., 2007).

### Functional significance of long-lasting AHP reduction

Unlike synaptic modifications, which can be restricted to specific synapses, a change in the neuron firing patterns would affect all its connections with other cells. Widespread modifications in neuronal excitability can be expected to increase the amount of information (expressed in the averaged action potential firing rate) processed in the network composed of these neurons. In particular, enhancement of intrinsic neuronal excitability results in enhanced learning abilities (Lisman et al., 2018; Chandra et al., 2019); for instance, enhanced intrinsic neuronal excitability enables neuronal ensembles to enter into a state, which may be best termed “*learning mode*”, by setting a time window in which activity-dependent synaptic modifications are more likely to occur as neurons are more easily recruited into new memory traces (Yiu et al., 2014; Gouty-Colomer et al., 2016; Chandra et al., 2019). Following OD rule learning, this state lasts in the piriform cortex for up to several days, and its behavioral manifestation is enhanced learning capability in complex tasks that require rule learning (Zelcer et al., 2006; Saar and Barkai, 2009; Chandra and Barkai, 2018).

### The illusive slow AHP channel

Although considerable efforts have been made to describe the molecular identity of the sI_AHP_ for the last 35 years, a specific channel through which the sI_AHP_ flows has not been identified (Andrade et al., 2012; King et al., 2015; Wang et al., 2016). An interesting study demonstrated that the functional expression of the sI_AHP_ in pyramidal cells is critically dependent on membrane phosphatidylinositol 4,5-bisphosphate [PtdIns(4,5)P(2)]. Moreover, PtdIns(4,5)P(2) regulates the calcium sensitivity of sI_AHP_ by acting downstream from the rise in intracellular calcium (Villalobos et al., 2011). These findings led to the interesting hypothesis that PtdIns(4,5)P(2) activates a variety of potassium channels (reviewed in Andrade et al., 2012). Thus, the sI_AHP_ would not represent a unitary ionic current but the embodiment of a generalized potassium channel gating mechanism (Andrade et al., 2012).

Here, we first show, using current-clamp recordings, that the post-burst AHP in piriform cortex pyramidal neurons is also mediated by the I_h_, which is also affected by learning but not by the IKCa current. We also show, using whole-cell patch-clamp recordings, that learning-induced reduction in the post-burst AHP results from a long-term reduction in the M muscarinic acetylcholine receptor.

## Methods

### Complex olfactory discrimination learning

#### Subjects

Age-matched young adult (~125–150 grams at the beginning of training) male Sprague Dawley rats were used. Before training, they were maintained on a 23.5-h water deprivation schedule, with food available *ad libitum*.

#### Apparatus and odors

**Figure 1:**
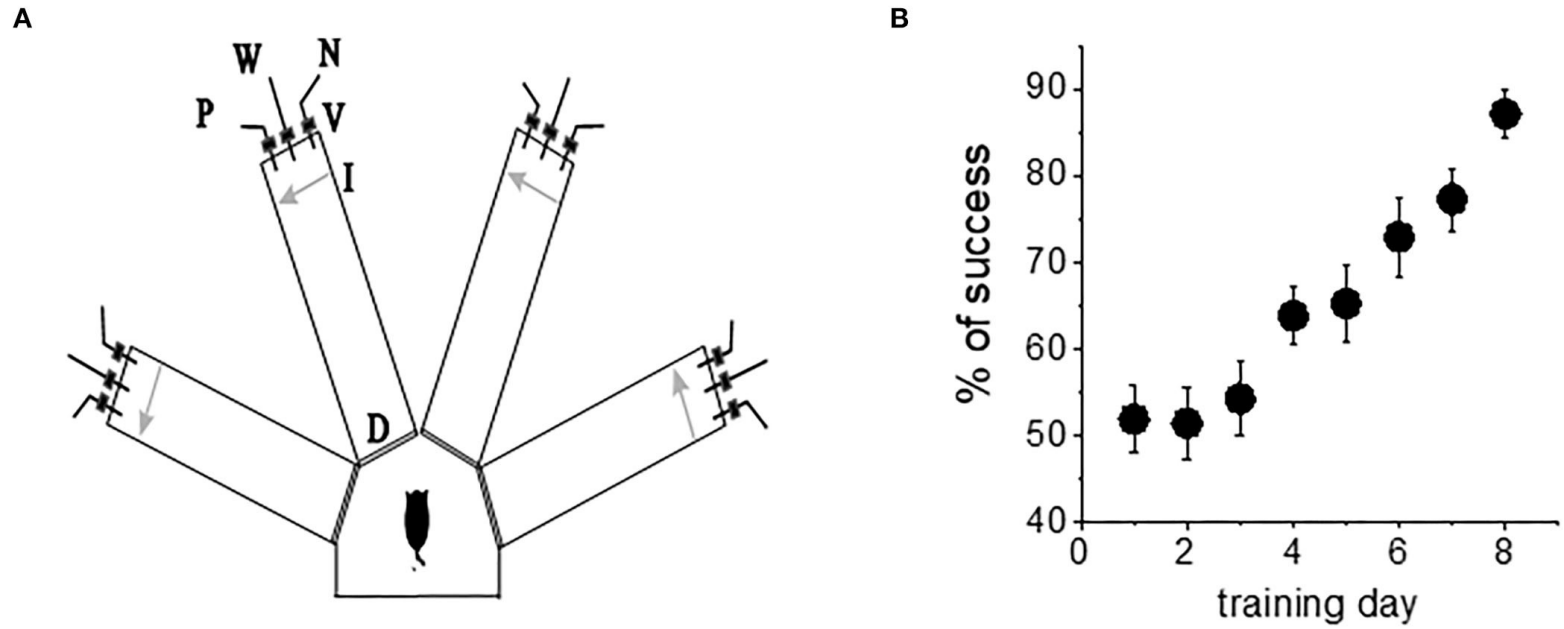
Complex olfactory learning apparatus and complex OD rule learning. **(A)** Similar protocols were applied for “trained” and “control” rats. An electronic “start” command randomly opens two of eight valves (V), releasing a positive-cue odor (P) into one of the arms and a negative-cue odor (N) into another. After 8 s, the two corresponding guillotine doors (D) are lifted to allow the rat to enter the selected arms. Upon reaching the far end of an arm (90 cm long), the rat body interrupts an infrared beam (I, arrow) and a drop of drinking water is released from a water hose (W) into small drinking well (for a “trained” rat—only if the arm contains the positive-cue odor, for “pseudo-trained” rat—randomly). A trial ends when the rat interrupts a beam, or in 10 s, if no beam is interrupted. A fan is operated for 15 s between trials, to remove odors. **(B)** Trained rats (*n* = 20) demonstrate acquisition of rule learning. With 20 trials per day, 8 consecutive days of training was required for this group to reach the criterion for discriminating between the first pair of odors (80% correct choices). Other groups usually require a similar period. Values represent mean ± SE.

The olfactory discrimination protocol was performed in a 4-arm radial maze, with commercial synthetic odors that are regularly used in the cosmetics and food industry purchased from Value Fragrances & Flavors. Odors were diluted by a factor of 1,000 before use in the olfactory maze. Odors were composed of natural and naturally derived materials in their formulations. The odors used in this study were as follows: almond, gardenia, wintergreen, and lavender (Figure 1A).

#### Training

Olfactory training consisted of 20 trials per day as previously described (Saar et al., 1998, 2001). The rats had to choose the correct odor (the odor which when chosen is awarded with drinking water). Learning was considered acquired upon demonstration of at least 80% positive-cue choices for the last 10 trials of the day. A pseudo-trained group of the age-matched rats was exposed to the same protocol of training but with random water rewarding. An age-matched naive group of rats was water-deprived but not exposed to any training. When trained in this complex task, the rats demonstrated increased capability to discriminate between new odors once they reach good performance with the first pair of odors (Saar et al., 1998, 2001). Notably, rule learning is acquired with great effort; it requires an average of 7–8 days of training for all rats to develop a strategy for performing the task and reach the criterion for rule learning (Figure 1B). As previously reported (Saar et al., 1998, 2001; Zelcer et al., 2006), the rats that learn the rule show enhanced capability to perform the same, as well as other complex tasks.

### *In vitro* physiological studies

#### Slice preparation and recordings

The rats were anesthetized using pentobarbital (30 mg/kg). Brain slices were taken from the posterior piriform cortex. The 400-μm coronal rat brain slices were used for intracellular sharp recordings, and 300-μm slices for whole-cell patch-clamp recordings were prepared as previously described (Saar et al., 1998, Saar et al., 2012) and kept for 1 h in oxygenated (95% O2 + 5% CO2) artificial cerebrospinal fluid (aCSF) solution (in mMol/L: NaCl 124, KCl 3, MgSO42, NaH2PO4 1.25, NaHCO3 26, CaCl2 2, and glucose 10). Intracellular recordings were obtained with 4 M K-acetate-filled sharp glass microelectrodes of 40–100 MΩ resistance, while the patch pipettes of 4–5 MΩ resistance were filled with internal solution (in mMol: K-gluconate 130, KCL 5, HEPES 10, EGTA 0.6, MgCl2 2.5, Mg-ATP 4, Na-GTP 0.4, and Na-phosphocreatine 10). Intracellular recordings with sharp electrodes were obtained as previously described (Cohen-Matsliah et al., 2007) using an Axoclamp 900A. Patch recordings were performed using an Axopatch 1D (Molecular Devices), and the data were acquired using a pClamp (Molecular Devices).

#### AHP and neuronal adaptation measurements with sharp electrodes

**Figure 2:**
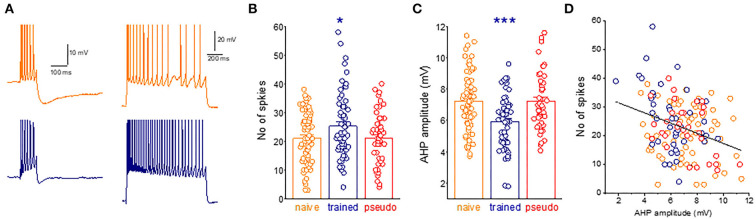
A widespread learning-induced decrease in the post-burst AHP amplitude mediates enhanced intrinsic neuronal excitability. **(A)** Examples for current-clamp recordings for AHPs and repetitive spike firing in neurons taken from naive (orange) and trained rat (navy) measurements in a piriform cortex neuron. For AHP recordings, the holding membrane potential is −60 mV, and the AHP is generated by a 100-ms depolarizing current step, with the intensity that generates a train of six action potentials. The presented trace is an average of five consecutive recordings made within intervals of 10 s. Repetitive spike firing is shown in the same neurons in response to intracellularly applied 1-s depolarizing, with a stimulus intensity of I_th_x2. **(B)** The graph shows the difference between groups in the number of spikes. Dots denote the spikes generated in each neuron. Values represent mean ± SE. *p < 0.05. Between-group comparison was done using one-way ANOVA for the three learning groups, with *post-hoc* multiple *t*-tests for each pair of groups. *Post-hoc* tests show that values of the train group differed significantly from values of the naive (*P* < 0.05) and the pseudo-trained group (*P* < 0.05). The two control groups did not differ between them (*p* = 0.95). **(C)** The difference between groups in the AHP amplitude. Dots denote the AHP value in each neuron. Values represent mean ± SE. ***p < 0.001. Between-group comparison was carried out using one-way ANOVA for the three learning groups, with *post-hoc* multiple *t*-tests for each pair of groups. *Post-hoc* tests show that values of the train group differed significantly from values of the naive (*P* < 0.001) and the pseudo-trained group (*P* < 0.001). The two control groups did not differ between them (*p* = 0.21). **(D)** The relation between the AHP amplitude and the number of action potentials generated by each neuron. Results are shown for 48 neurons. A highly significant negative correlation between the AHP amplitude and the number of action potentials is apparent (*r* = −0.32, *p* < 0.001).

AHPs were recorded within minutes after good recording conditions were established [resting potential of at least 65 mV and action potential (AP) amplitude of 80 mV]. For post-burst AHP measurement, neurons were depolarized in the current-clamp mode to a holding potential of −60 mV with direct current injection *via* a recording electrode, and 100-ms-long depolarizing current steps were applied with intensity sufficient to evoke trains of six action potentials. AHP amplitude was measured from baseline to the peak of the hyperpolarizing voltage deflection that follows the spike train (Figure 2A). The AHP value was determined from the averaged amplitude in four or five consecutive traces, evoked at intervals of 10 s. For neuronal adaptation measurements, a prolonged (1 s) depolarizing current step was applied to the neurons *via* a recording electrode at V_rest_, with a stimulus intensity of a 2-fold I_Threshold_ (Figure 2A).

#### Voltage clamp recordings of AHP currents

For direct measurement of the currents that underlie the AHP, whole-cell gigaseal voltage-clamp recordings were obtained from layer II pyramidal neurons and visualized by infrared DIC video microscopy. Recordings were performed at room temperature. The intrinsically evoked sI_AHP_ was recorded in a TTX (1 μM) and TEA (5 mM) containing aCSF solution (**Figure 4A**). To generate a pure sI_AHP_, apamin (50 nM), the SK channel blocker was added to the solution (Brosh et al., 2006). Neurons were kept at a holding potential of −60 mV, and a 200-ms depolarizing pulse was applied to elicit an unclamped calcium action current, evoking the AHP currents recorded in the voltage clamp. The peak amplitude and duration of the post-depolarization currents were measured after such a standard depolarizing pulse. The effect of prolonged recording on the sI_AHP_ rundown was determined by calculating the percent change in the peak amplitude and duration.

### Statistical analysis

Between-group comparison was carried out using one-way ANOVA for the three groups (naive, trained, and pseudo-trained), and *post-hoc* multiple *t*-tests were then applied to compare between two groups. The effect of recording time and drug application on the sI_AHP_ and the post-burst AHP amplitude decline was examined for each neuron with and without the treatment, using a paired *t*-test. The paired *t*-test was also applied when comparing the AHP amplitude in the same neurons before and after drug application. An unpaired *t*-test was applied when comparing neurons from two different experimental groups under different conditions. Values throughout the text are presented as mean ± SD. Data in graphs are presented as mean ± SE.

## Results

### Complex OD learning-induced enhanced intrinsic excitability is mediated by a reduction in the post-burst afterhyperpolarization

We previously showed that rule learning-induced post-burst AHP reduction is mediated by metabotropic activation of GluK2 (kainate receptor), and that GluK2 is both necessary and sufficient for OD rule learning (Chandra et al., 2019). We showed that GluK2 knockout mice are incapable of learning the complex OD task, although their ability to learn simple olfactory tasks (such that do require learning of a rule) remains intact. Moreover, viral-induced overexpression of Gluk2 in piriform cortex pyramidal neurons enhances rule learning remarkably. Thus, a causal relationship can be established between AHP reduction and rule learning.

The next necessary step is to show that the post-bust AHP reduction is indeed a dominant factor in increasing repetitive spike firing, evoked by activation of intrinsic neuronal currents. The average number of action potentials evoked by a prolonged intrinsically applied depolarizing current pulse in neurons from trained rats (25.2 ± 11.6, *n* = 61) was significantly higher (*p* = 0.032, F = 3.5) than that in neurons from the naive (21.0 ± 9.1, *n* = 71) and pseudo-trained (21.1 ± 9.2, *n* = 49) rats (Figures 2A,B).

As shown before (Saar et al., 1998, 2001; Cohen-Matsliah et al., 2007; Chandra and Barkai, 2018), the average amplitude of the post-bust AHP evoked by repetitive spike firing in neurons from trained rats (5.9 ± 1.7 mV, *n* = 60) was significantly lower (*p* = 0.00044, F = 10.2) than that in neurons from the naive (7.2 ± 1.9, *n* = 71) and pseudo-trained (7.2 ± 1.8, *n* = 47) rats (Figure 2C).

The strong correlation between the post-burst AHP and the number of intrinsically evoked action potentials shown in Figure 2D suggests that learning-induced enhanced neuronal excitability is strongly related to the decreased post-burst AHP in these neurons.

### The post-burst AHP is not mediated by the calcium-dependent potassium currents, I_AHP_, and SI_AHP_, only

As noted before, ample evidence suggests that the post-burst AHP reduction in piriform cortex pyramidal neurons results from decreased conductance of the sI_AHP_. Specifically, this notion is supported by three main findings: *a*. The difference between neurons from the trained and control mice in the post-burst AHP amplitudes is abolished in the presence of an intrinsically applied calcium chelator (Saar et al., 2001). *b*. By the application of apamin, the specific I_AHP_ agonist does not abolish the difference in the post-burst AHP amplitude between neurons from the trained and control animals. In fact, it enhances it (Brosh et al., 2006). *c*. The reduction in post-burst AHP amplitude is not accompanied by a change in its reversal potential, which would indicate that another non-potassium current has been modified (Saar et al., 2001). Thus, although the channel that mediates the sI_AHP_ was never identified and the current could not be specifically blocked, it was deduced that it is the current which is blocked by rule learning.

Notably, all the aforementioned experiments were performed in neurons recorded with sharp intracellular electrodes, in current-clamp mode. This technique allowed prolonged stable recordings of the AHP, for many tens of minutes (Chandra et al., 2019), avoiding the washout of the current, which occurs while recording with whole-cell patch-clamp electrodes. However, current-clamp recordings do not reveal the influence that overlapping currents may have on each other, through voltage-dependent effects. In other words, while it is clear that changes in the sI_AHP_ play a key role in learning-induced enhanced intrinsic excitability, the possibility remains that the contribution of other currents, not necessarily calcium-dependent currents, to the post-burst AHP are also modified by learning.

**Figure 3:**
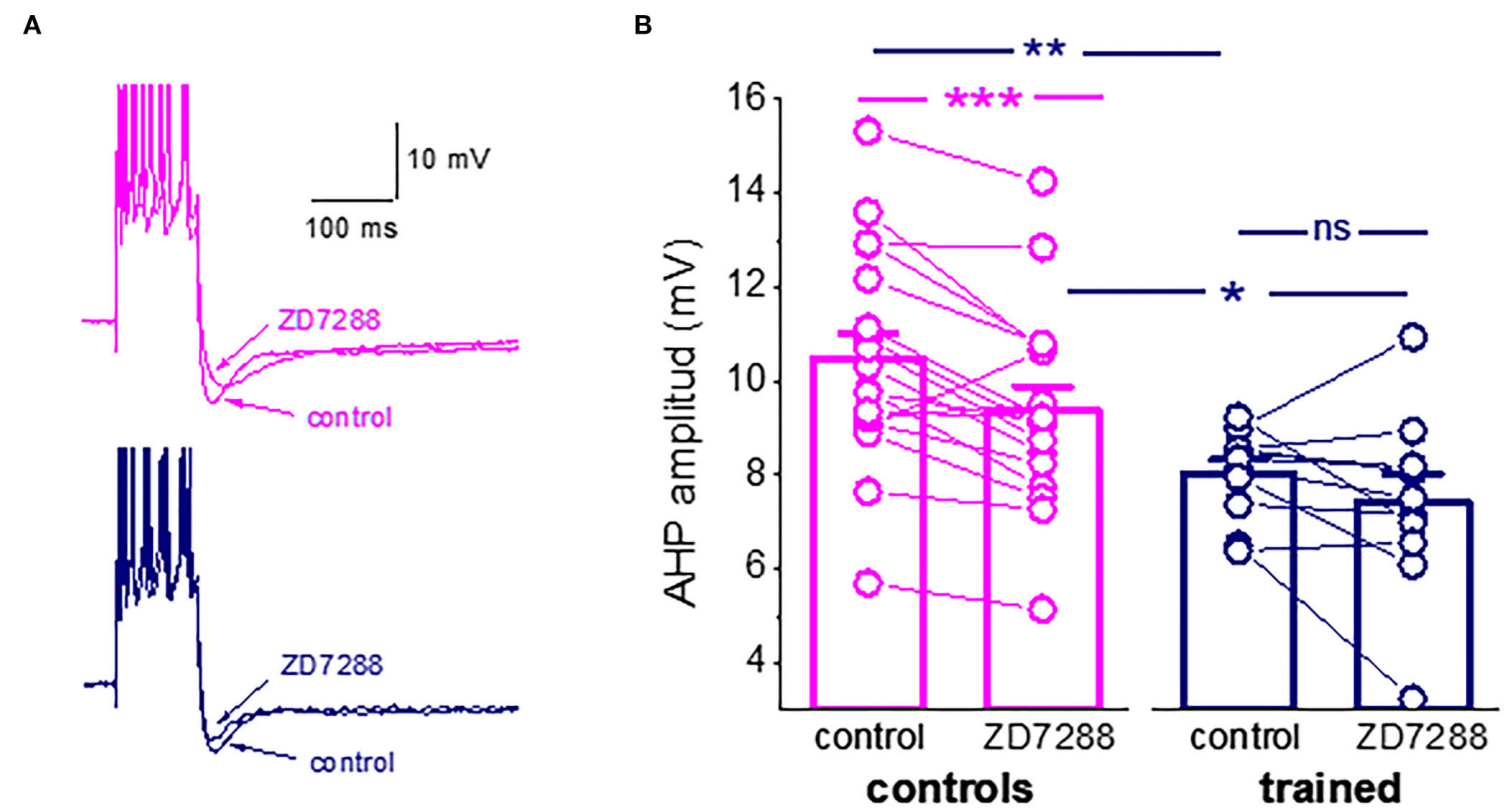
I_h_ is active during the post-Burst AHP in neurons from control rats. **(A)** Averaged traces from pseudo-trained and trained neurons, before and 20 min after the ZD7288 application. The I_h_ blocker reduced the post-burst AHP in the pseudo-trained neuron only. **(B)** The I_h_ blocker, ZD7288, reduces the post-burst AHP amplitude in neurons from the controls, but not from the trained rats. Bars represent mean ± SE. **p < 0.051. *p < 0.05. A paired *t*-test was used to compare the effect of ZD7288 of each group of neurons. An unpaired *t*-test was used to examine the difference between neurons from the trained and control rats. *p < 0.05; **p < 0.01; ***p < 0.001; ns, not significant.

One particularly interesting intrinsic potassium current shown to be involved in the learning process is the hyperpolarization-activated mixed cation current, I_h_. The I_h_ has been shown to regulate neuronal excitation by altering the size and kinetics of the AHP (Storm, 1990; MacCaferri et al., 1993; Poolos et al., 2002; Gu et al., 2005). The current is activated with hyperpolarization and deactivates with depolarization. The HCN channel that mediates this current has permeability to K^+^ and Na^+^ ions, resulting in a reversal potential of approximately −30 mV, causing these channels to generate an excitatory inward current. We then tested the effect of the I_h_ blocker, ZD7288 (25 μM), on the post-burst AHP amplitude. The I_h_ blocker significantly reduced the amplitude of the average post-burst AHP in neurons for control rats (from 10.5 ± 2.3 mV to 9.4 ± 2.1 mV (*n* = 18, *p* = 0.0007). Such an effect was not observed in neurons from trained rats (from 8.04 ± 0.9 mV to 7.4 ± 1.9 mV (*n* = 11, *p* = 0.19). Notably, the difference in the average post-burst AHP between neurons from trained rats and controls remained significant in the presence of the blocker (Figures 3A,B).

These results should not be interpreted as evidence for a learning-induced reduction in the I_h_. It may well be that it is not modified by learning. However, the reduction in the AHP amplitude may result from less activation of this hyperpolarization-activated current, which would in turn affect intrinsic neuronal excitability. Thus, the composition of currents that generate the post-burst AHP is far more complex than previously described. To more precisely identify the currents that are reduced in piriform cortex pyramidal neurons, we tested how OD learning affects the different components of the sI_AHP_, using prolonged whole-cell patch-clamp recordings, during which the current decays as a function of the recording time.

### Learning-induced reduction in the slow AHP mediated by changes in the continuous activation of PtdIns(4,5)P(2)

**Figure 4:**
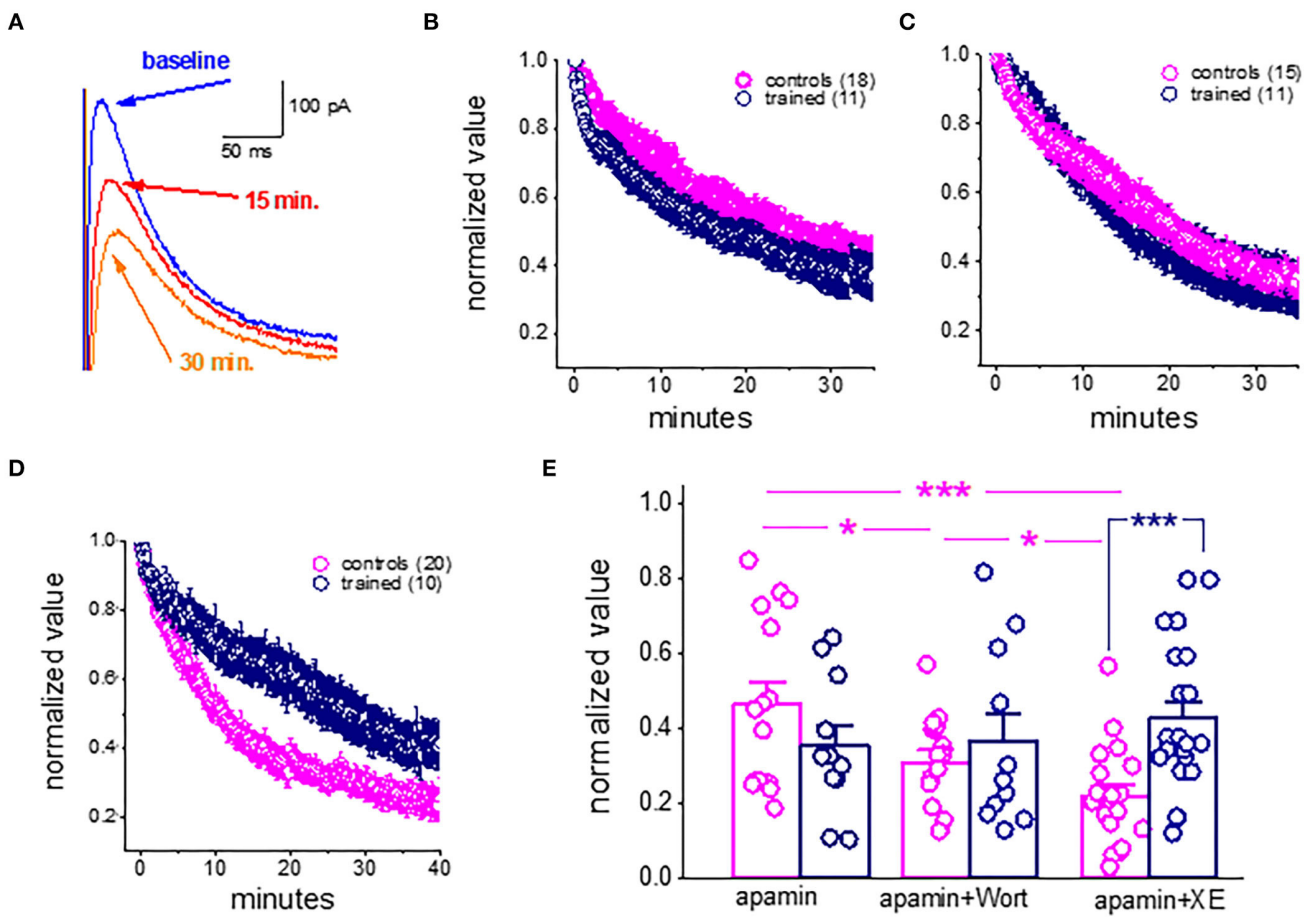
Rundown of the sI_AHP_ upon prolonged recordings shows that the M current is not active in piriform cortex pyramidal neurons after learning. **(A)** Whole-cell voltage-clamp recordings from a pyramidal neuron. The cell was voltage-clamped at −60 mV. A 200-ms depolarizing pulse to +60 mV generated an unclamped calcium current (truncated). After its termination, an outward current appears with a reversal potential of −80 mV. Traces illustrating the rundown of the sI_AHP_. The blue trace shows the sI_AHP_ when the stable recording was established. Red and orange traces were taken after 15 and 30 min. **(B)** Summary plot illustrating the rundown of the sI_AHP_ in neurons from trained and control rats. Recordings were carried out in the presence of the SK channel blocker apamin, to ensure that only the sI_AHP_ is activated. **(C)** Rundown of the sI_AHP_ in the presence of apamin and wortmannin, to inhibit PtdIns(4,5)P(2) biosynthesis. **(D)** Rundown of the sI_AHP_ in the presence of apamin and XE991, to block the cholinergic M current. **(B,D)** values represent mean ± SE, and *n* denotes the numbers of neurons. **(E)** Bars show the average normalized decline for each treatment. In apamin only, there was no difference in the rundown kinetics between the controls and trained neurons. The presence of wortmannin enhanced the sI_AHP_ rundown in neurons from controls only. Application of the M current blocker greatly enhanced the sI_AHP_ decay in neurons from controls only. Notably, both treatments had no effect on the sI_AHP_ decay in neurons from trained rats. *p < 0.05. ***p < 0.001. The paired *t*-test was used to compare the effect of drug application on each group of neurons. The unpaired *t*-test was used to examine the difference between neurons from trained and control rats.

As previously shown for cortical neurons (Villalobos et al., 2011), during prolonged (tens of minutes) continuous sI_AHP_ recordings using whole-cell patch-clamp electrodes, the sI_AHP_ in piriform cortex pyramidal amplitude declines steadily and is reduced to about 40% of its initial values within 30 min (Figures 4A,B). As noted previously, this rundown of the I_AHP_ is strongly dependent on PtdIns(4,5)P(2); facilitation of PtdIns(4,5)P(2) biosynthesis protects against sI_AHP_ rundown, and inhibition of its biosynthesis accelerates the sI_AHP_ rundown (Villalobos et al., 2011).

We thus examined if the rundown of the sI_AHP_ differs between neurons taken from the trained rats and controls and if inhibition of PtdIns(4,5)P(2) biosynthesis affects this rundown differently.

Our results show that in control conditions (with only apamin in the aCSF solution), the rundown of the sI_AHP_ is similar in neurons from the trained rats and controls (Figure 4B). The averaged normalized value of the 30–35 min after the beginning of recordings was 0.44 + 0.23 (*n* = 15) for neurons from controls and 0.36 + 0.19 (*n* = 11) for neurons from trained rats (*P* = 0.39) (Figure 4E). When recording in the presence of the PtdIns(4,5)P(2) biosynthesis inhibitor, wortmannin (10 nM) (Figures 4C,E), the normalized values were 0.31 + 0.10 (*n* = 15) for neurons from controls and 0.37 + 0.24 (*n* = 11) for neurons from trained rats (*p* = 0.44). Notably, while wortmannin significantly reduced the normalized value in neurons from controls (*P* < 0.05, Figure 4E), it did not affect the averaged value in neurons from the trained rats (*p* = 0.89).

Thus, while blocking PtdIns(4,5)P(2) biosynthesis in piriform cortex pyramidal neurons has a similar effect to that described for cortical neurons (Villalobos et al., 2011), it did not have any impact on the amplitude of neurons from trained rats. These results suggest that the currents which are PtdIns(4,5)P(2) activation-dependent are the ones that are reduced in pyramidal neurons after learning.

### The M receptor-mediated potassium current is reduced after learning

We previously showed that after rule learning, the cholinergic agonist, carbachol, loses its ability to reduce the AHP in piriform cortex neurons, and in parallel, blocking cholinergic activity by injecting scopolamine does not disrupt olfactory learning once the rule was acquired (Saar et al., 2001).

We thus tested the effect of XE991 dihydrochloride, the potent K_v_7 (KCNQ) channel blocker which is specific to the cholinergic M current. XE991 (10 μM) had a profound effect on the rundown of the sI_AHP_ in neurons from the controls (Figures 4D,E). The averaged value of the normalized AHP in neurons from controls at the time interval of 30–35 min was 0.21 ± 0.13 (*n* = 19). This value is significantly lower than the averaged values in apamin and wortmannin (*p* < 0.05) (Figure 4E). In sharp contrast, application of the XE991 had no effect on the averaged normalized value in neurons from the trained rats (0.43 ± 0.2 (*n* = 21); it remained similar to that obtained in the presence of apamin only (*p* = 0.30), and in the presence of apamin+wortmannin (*p* = 0.45). Notably, in the presence of XE991, the averaged values in neurons from the trained rats were significantly higher than those in controls (*p* < 0.001) (Figure 4E).

Taken together with our previous findings (Saar et al., 2001, see **Figure 7**), these results suggest that the shutdown of the M receptor-mediated current enables prolonged OD learning-induced reduction of the post-burst AHP current.

### Current-clamp recordings reveal that blocking PtdIns(4,5)P(2) biosynthesis does not affect the post-burst AHP amplitude

Based on the results of the previous voltage-clamp recordings, we hypothesized that the rule learning induced reduction in the M-current conductance and thus in the post-burst AHP is mediated by reduction of the current by modulation of PtdIns(4,5)P(2) activity. To test this hypothesis, we measured the effect of the PtdIns(4,5)P(2) biosynthesis inhibitor, wortmannin, on the post-burst AHP amplitude recorded under current-clamp conditions on neurons from the control and trained rats. We predicted that wortmannin would reduce AHP in neurons from the control rats only.

**Figure 5:**
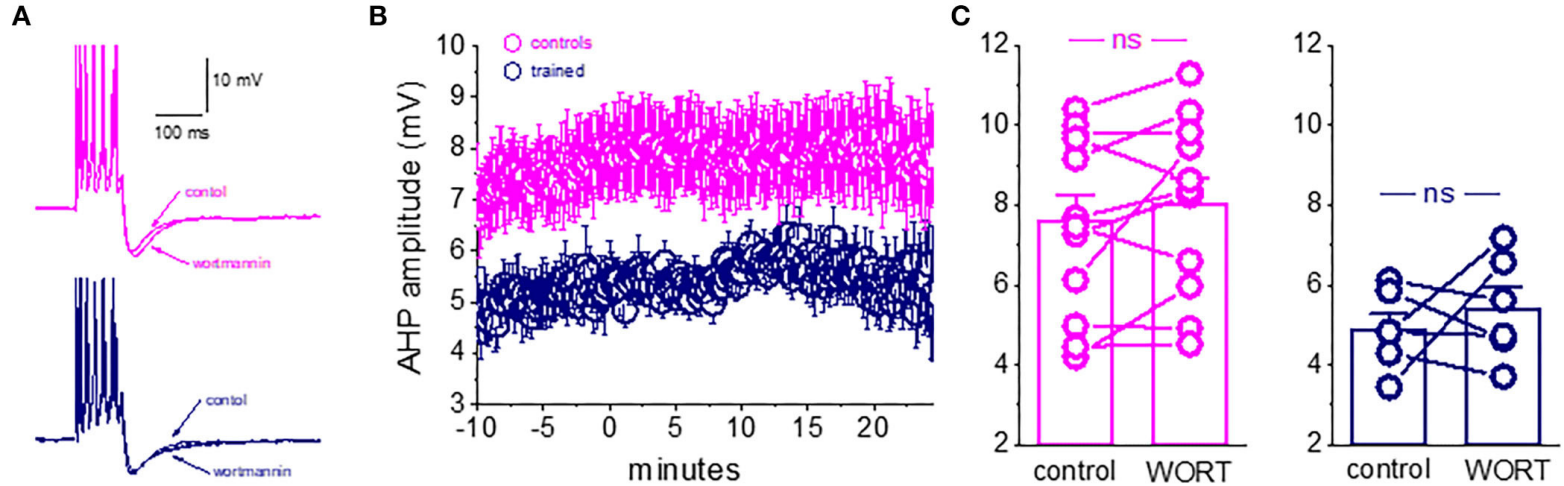
Blocking PtdIns(4,5)P(2) biosynthesis does not affect the post-burst AHP amplitude. **(A)** Averaged traces from pseudo-trained and trained neurons, before and 20 min after wortmannin application. Wortmannin had no effect on the post-burst AHP in both neurons. **(B)** Timeline of continuous AHP recordings before (−10 to 0 min) and after wortmannin application in neurons from control and trained rats. Values represent mean ± SE. **(C)** Direct comparison of the AHP values in each cell before and 20 min after wortmannin application in neurons from control and trained rats. Wortmannin did not change the post-burst AHP amplitude in controls or trained neurons. Values represent mean ± SE. The paired *t*-test was used to compare the effect of wortmannin on each group of neurons. ns, not significant.

We find that wortmannin had no effect on the post-burst AHP in piriform cortex pyramidal neurons. As shown in Figure 5, the application of wortmannin for tens of minutes did not change the post-burst AHP amplitude in neurons from the control or trained rats. The post-burst AHP remained stable throughout the recording period, before and after wortmannin application (Figures 5A,B). In 11 neurons from the control rats, the averaged post-burst AHP amplitude was 7.3 ± 2.2 mV before and 8.0 ± 2.2 mV after 20 min of wortmannin application (*p* = 0.12). In six neurons from the trained rats, the averaged post-burst AHP amplitude was 4.9 ± 1.0 mV before and 5.4 ± 1.3 mV after 20 min of wortmannin application (*p* = 0.51). Consequently, as shown in Figure 5C, the significant difference in amplitudes between neurons from the control and trained rats in control conditions (*p* = 0.013, *t* = 2.78) remained after wortmannin application (*p* = 0.02, *t* = 2.59).

These data do not support a role for modulation of the PtdIns(4,5)P(2)-mediated process in maintaining long-lasting AHP reduction in piriform cortex neurons after complex olfactory learning.

### The intermediate conductance calcium-activated potassium channels (IKCa) do not have a role in the post-burst ahp in piriform cortex pyramidal neurons

Previous work suggests that the IKCa channels have a role in generating the sAHP of CA1 hippocampal pyramidal cells (Turner et al., 2016). We examined if this current is also involved in the generation of post-burst AHP in piriform cortex pyramidal neurons. To examine this possibility, we applied the IKCa channel blocker TRAM-34.

**Figure 6:**
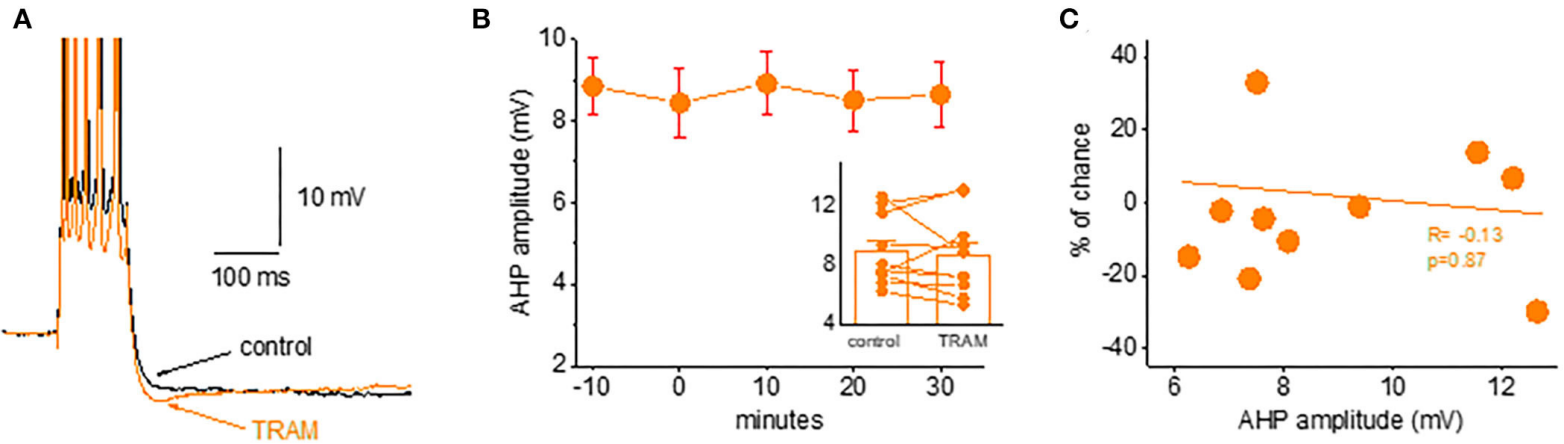
Blocking the *IKCa* channels does not affect the post-burst AHP amplitude. **(A)** Averaged traces from a naive neuron before and 30 min after TRAM-34 application. The IKCa blocker had no effect on the post-burst AHP. **(B)** Timeline of continuous AHP recordings before (−10 to 0 min) and after TRAM-34 application in neurons from control and trained rats. Values represent mean ± SE. *Inset*: Direct comparison of the AHP values in each cell before and 30 min after TRAM-34 application. Values represent mean ± SE. **(C)** The relation between the initial post-burst AHP amplitude and the effect of TRAM-34 in each neuron. TRAM-34 did not affect the AHP amplitude, regardless of its initial value (*r* = −0.13, *p* = 0.87). The paired *t*-test was used to compare the effect of TRAM.

We found that TRAM-34 (1 μM) had no effect on the post-burst AHP in piriform cortex pyramidal neurons. As shown in Figure 6, the application of TRAM-34 for 30 min did not change the post-burst AHP amplitude in neurons from naive rats. The post-burst AHP remained stable throughout the recording period, before and after TRAM-34 application (Figures 6A,B). In 10 neurons, the averaged post-burst AHP amplitude was 8.9 ± 2.4 mV before and 8.7 ± 2.8 mV after 30 min of TRAM-34 application (*p* = 0.62). The lack of effect of TRAM-34 on the post-burst AHP was apparent in all recorded neurons, regardless of the initial post-burst AHP measured in each neuron (Figure 6C).

These data do not support a role for IKCa channels in generating the post-burst AHP in piriform cortex neurons.

## Discussion

In this study, we show that while the post-burst AHP appears to be mediated by a complex combination of overlapping, calcium-dependent, and voltage-dependent currents, the shutdown of the M-receptor-mediated current is the principal mechanism underlying the learning-induced enhancement of neuronal excitability.

### Learning-induced enhancement in neuronal excitability

Learning-induced enhancement in neuronal excitability has been shown in hippocampal neurons following classical conditioning of the trace eyeblink response (Moyer et al., 1996; Thompson et al., 1996) and the Morris water maze task (Oh et al., 2003), and in piriform cortex neurons following operant conditioning (Saar et al., 1998, 2001; Saar and Barkai, 2003). Olfactory discrimination learning also results in enhanced neuronal excitability in CA1 hippocampal (Zelcer et al., 2006) and basolateral amygdala (Motanis et al., 2014) neurons. Such enhanced excitability is expressed in reduced spike frequency adaptation in response to prolonged depolarizing current applications (Moyer et al., 1996; Thompson et al., 1996; Saar et al., 2001).

Neuronal adaptation in hippocampal and cortical pyramidal neurons is modulated by medium and slow afterhyperpolarization (AHPs), generated by potassium currents, which develop following a burst of action potentials (Madison and Nicoll, 1984; Constanti and Sim, 1987; Schwindt et al., 1988; Saar et al., 2001). Indeed, it was shown in hippocampal and piriform cortex pyramidal neurons that the post-burst AHP amplitude is reduced after OD rule learning (Moyer et al., 1996; Saar et al., 1998; Zelcer et al., 2006).

Our findings show a clear correlation between the post-burst AHP amplitude and the number of action potentials generated by intrinsically applied prolonged depolarization. However, while the intuitively learning-induced reduction in the medium and late AHPs should indeed be expressed in the neuron ability to sustain repetitive spike firing, it should be noted that the relations between AHP reduction and intrinsic neuronal adaptation are not that simple. For example, learning-induced AHP reduction is accompanied by a shutdown of the I_h_. Since this current enhances neuronal excitability (Storm, 1990; MacCaferri et al., 1993; Poolos et al., 2002; Gu et al., 2005), its learning-induced shutdown may result in reduced neuronal excitability. This finding further testifies to the complexity of the relations between learning-induced post-burst AHP reduction and enhanced intrinsic excitability.

### Learning-induced AHP reduction is mediated at least practically by the block of the M-receptor-mediated current

The inability to identify the sI_AHP_ channel has puzzled many researchers in the last decade (Andrade et al., 2012; King et al., 2015; Wang et al., 2016). An intriguing solution to this puzzle was suggested by Andrade and his collogues (Andrade et al., 2012). Based on results obtained by them (Villalobos and Andrade, 2010; Villalobos et al., 2011) and others, they suggested that the sI_AHP_ differs from other calcium-dependent currents that mediate the early and medium AHPs in that it appears to sense cytoplasmic [Ca(2+)] and that neuronal calcium sensors are mediators for the sI_AHP_. The sI_AHP_ is carried by different potassium channel types in different cell types (Andrade et al., 2012). Importantly, the sI_AHP_ is dependent on membrane PtdIns(4,5)P(2), and Ca(2+) appears to gate this current by increasing PtdIns(4,5)P(2) levels, which in turn activates a variety of potassium channels. Thus, the sI_AHP_ does not represent a single current but is the embodiment of a generalized potassium channel gating mechanism.

Based on their model, we first examined if inhibiting PtdIns(4,5)P(2) biosynthesis would have a different effect on the decay rate of the sI_AHP_ during recording piriform cortex pyramidal neurons with whole-cell patch-clamp electrodes. We found that blocking PtdIns(4,5)P(2) biosynthesis significantly enhances the decay time course of the sI_AHP_ in neurons from the control rats only, but it did not affect neurons from the trained rats. However, application of the same blocker had no effect on the post-burst AHP amplitude, recorded with sharp intracellular electrodes, in neurons from the controls, as well as from the trained rats. Thus, while leaning-induced modifications in the sI_AHP_ are accompanied by changes in its sensitivity to PtdIns(4,5)P(2), the post-burst AHP in the same neurons shows no response to the same blocker. A similar lack of effect on the AHP was found for the intermediate conductance calcium-activated potassium channels (IKCa).

**Figure 7:**
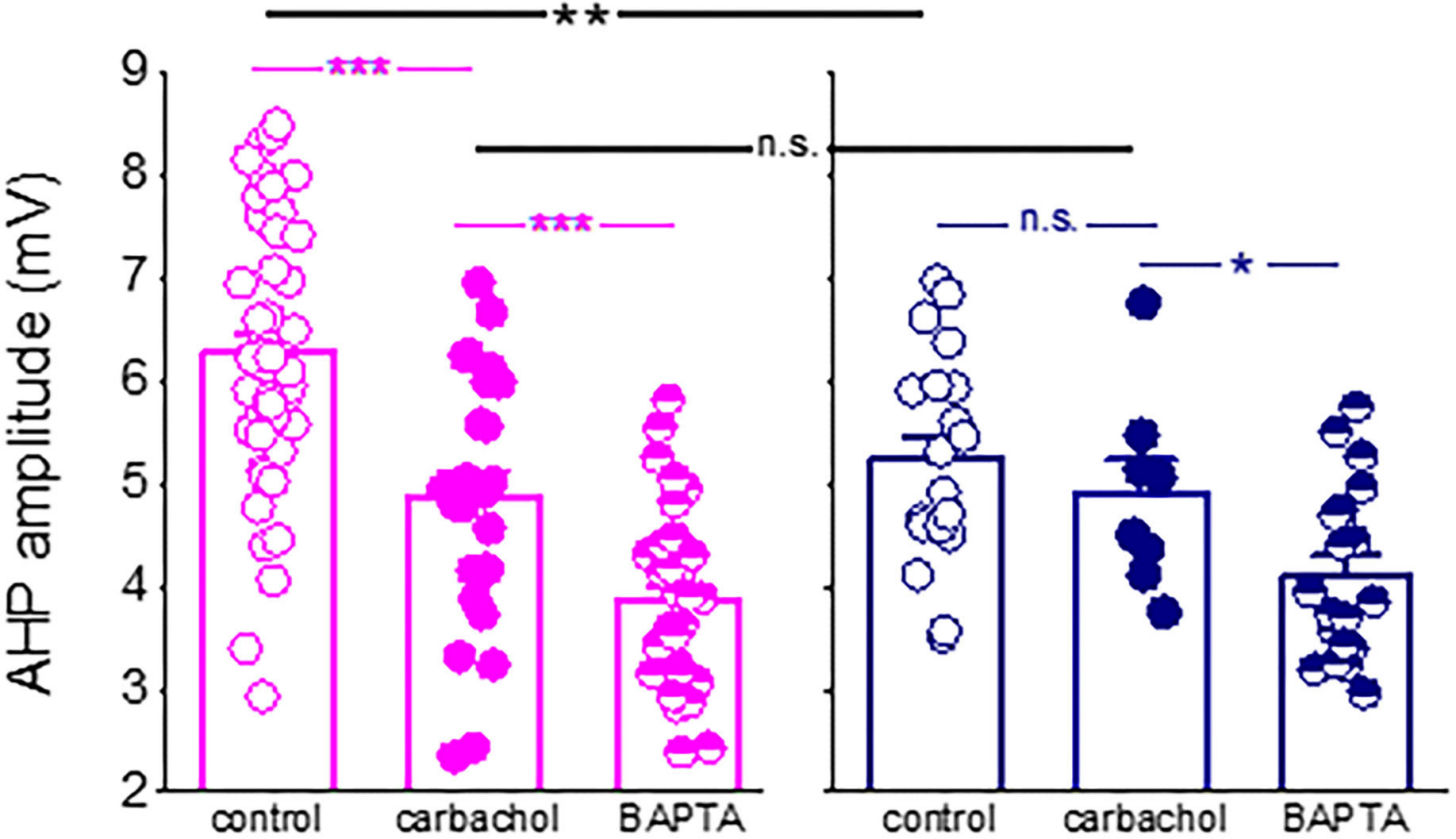
Difference in the post-burst AHP amplitude between neurons from control and trained rats is abolished in the presence of a cholinergic agonist. Application of the cholinergic agonist, carbachol, reduced significantly the post-burst AHP amplitude in neurons from control rats (*P* < 0.001) but did not affect the post-burst AHP in neurons from trained rats. Moreover, in the presence of the agonist, the difference in the post-burst AHP amplitude between the two groups (*p* < 0.01) was abolished. Notably, intracellular application of the calcium chelator BAPTA *via* the recording electrode (Saar et al., 2001) further reduced the post-burst AHP in neurons from control and trained rats. Bars represent mean ± SE. Each point reorients the post-burst AHP value in a cell. All data are taken from our previous publication (Saar et al., 2001). The unpaired *t*-test was used to examine the difference between neurons from trained and control rats and between neurons from the same groups at different conditions. *p < 0.05; **p < 0.01; ***p < 0.001; n.s., not significant.

We also found that while blocking the M-current significantly enhanced the decay of the sI_AHP_ in neurons from the control rats only, it had no effect on neurons from the trained rats. These data complete well the results from our previous study (Saar et al., 2001), in which we showed that the cholinergic agonist carbachol reduced both the post-burst AHP and firing adaptation in neurons from control rats but had no effect on neurons from the trained rats, suggesting a pre-existing cholinergic effect (Figure 7). These data also suggest that the post-burst AHP is mediated by an additional calcium-dependent potassium current. However, these other currents are not modified by learning, at least in a manner that would underlie the rule learning-induced modulation of the AHP and intrinsic neuronal excitability.

To conclude, our data show that OD learning-induced reduction in the post-burst AHP in piriform cortex pyramidal neurons is strongly correlated with the ability of these neurons to sustain repetitive spike firing, as expected. While the post-burst AHP is a complex phenomenon, composed of several currents, and sensitive to different neuromodulators and neuropeptides, rule learning-induced long-lasting reduction of the post-burst AHP is mediated, at least mostly, by reduced conductance of the cholinergic M-current.

## Data availability statement

The raw data supporting the conclusions of this article will be made available by the authors, without undue reservation.

## Ethics statement

The animal study was reviewed and approved by University of Haifa Ethics Committee.

## Author contributions

RA and NC performed the experiments and analyzed the data. EB planned the experiments and wrote the manuscript. All authors contributed to the article and approved the submitted version.

## Funding

This study was supported by a grant from the Israel Science Foundation to EB.

## Conflict of interest

The authors declare that the research was conducted in the absence of any commercial or financial relationships that could be construed as a potential conflict of interest.

## Publisher's note

All claims expressed in this article are solely those of the authors and do not necessarily represent those of their affiliated organizations, or those of the publisher, the editors and the reviewers. Any product that may be evaluated in this article, or claim that may be made by its manufacturer, is not guaranteed or endorsed by the publisher.
